# Effect of Tirofiban Injection on vascular endothelial function, cardiac function and inflammatory cytokines in patients with acute myocardial infarction after emergency Percutaneous Coronary Intervention

**DOI:** 10.12669/pjms.38.1.4413

**Published:** 2022

**Authors:** Shi-xin Kang, Xiao-min Meng, Jing Li

**Affiliations:** 1 Shi-xin Kang, Department of Cardiology, Affiliated Hospital of Hebei University, Baoding 071000, Hebei, China; 2 Xiao-min Meng, Department of Cardiology, Affiliated Hospital of Hebei University, Baoding 071000, Hebei, China; 3 Jing Li, Department of Cardiology, Affiliated Hospital of Hebei University, Baoding 071000, Hebei, China

**Keywords:** After emergency PCI, Tirofiban injection, Vascular endothelial function, Cardiac function, Treatment

## Abstract

**Objectives:**

To evaluate the effect of tirofiban injection on vascular endothelial function, cardiac function, inflammatory cytokines and other indicators in patients with acute myocardial infarction after emergency PCI and its clinical significance.

**Methods:**

Eighty patients with acute myocardial infarction admitted to Affiliated Hospital of Hebei University from March 18, 2020 to October 18, 2020 were enrolled and randomly divided into two groups: the experimental group and the control group, with 40 cases in each group. Patients in both groups underwent PCI. Patients in the control group were given oxygen inhalation, monitoring, and basic medications for myocardial infarction, such as nutritional myocardial drugs, statins, aspirin, nitrates, clopidogrel, and β-blockers. In contrast, patients in the experimental group received tirofiban 10 ug/kg intravenously over 5min immediately before PCI in addition to basic treatment, and then tirofiban 0.1 ug/(kg/min) was pumped via intravenous pump postoperatively for 48 hour. The changes of vascular endothelial function, cardiac function and adverse drug reactions (ADRs) in the two groups before treatment, one week and one month after treatment, as well as changes of inflammatory cytokines such as CRP and IL-6 in the two groups before and after treatment were compared and analyzed.

**Results:**

Compared with the control group, FMD, NO, ET-1 and other indexes in the experimental group were significantly improved one week and one month after treatment, with statistically significant differences (*p*<0.05). BNP, LVEDD, LVEF and additional indexes in the experimental group were significantly lower than those in the control group at one week and one month after treatment, with statistically significant differences (*p*=0.00). Moreover, the incidence of ST-segment fallback > 70% in the experimental group was 72.5% after treatment, which was significantly better than that of 47.5% in the control group, with a statistically significant difference (*p*=0.03). CRP and IL-6 in the experimental group were significantly lower than those in the control group after treatment, with a statistically significant difference (*p*=0.00). There was no statistical significance in the incidence of ADRs between the two groups after treatment (*p*=0.42).

**Conclusion:**

Tirofiban injection after emergency PCI is a beneficial treatment regime for patients with STEMI. With such a treatment regime, cardiac function and vascular endothelial function of patients can be dramatically improved, coronary blood supply will be ameliorated, inflammatory cytokines can be reduced, and no significant increase can be seen in the incidence of adverse reactions.

## INTRODUCTION

Acute myocardial infarction (AMI), as one of the most common cardiovascular diseases in middle-aged and elderly patients,^1^ can be ascribed to coronary atherosclerosis that causes coronary artery stenosis or occlusion, leading to acute myocardial ischemia and even necrosis.^2^ For patients suffering from AMI, the pumping function and conduction system of the heart are affected, leading to cardiac insufficiency, pulmonary edema, various arrhythmias, and in severe cases, cardiac arrest.^3^ The pathophysiological basis of ST-segment elevation myocardial infarction (STEMI) is mainly the transmural injury of the myocardial wall, and its treatment focuses on timely restoration of coronary blood flow and antithrombotic therapy.^4^ Percutaneous coronary intervention (PCI) has been clinically adopted as the main treatment scheme for STEMI, which is characterized by its remarkable efficacy, rapid onset, improved protection of patients’ cardiomyocytes, prevention of further ischemic injury, and reconstruction of coronary blood supply.^5^ However, certain shortcomings can be apparent in such a treatment regimen, such as the pressure of the coronary vessel wall caused by the surgical instruments, and the occurrence of microcirculation embolism postoperatively, which reduces the postoperative therapeutic activity. Tirofiban is a glycoprotein IIb/IIIa inhibitor with a potent antiplatelet therapy that has been shown to reduce cardiac ischemia complications in patients undergoing PCI.^6^ Lot of benefits can be derived from tirofiban injection after emergency PCI. For example, cardiac function and vascular endothelial function can be considerably improved, coronary blood supply will be ameliorated, and inflammatory cytokines can be reduced. The specific details are reported as following:

## METHODS

Eighty patients with acute myocardial infarction admitted to Affiliated Hospital of Hebei University from March 18, 2020 to October 18, 2020 were enrolled and randomly divided into two groups: the experimental group and the control group, with 40 cases in each group. Among the 80 patients, 26 males and 14 females were grouped into the experimental group, aged 55-71 years old, with an average of 60.15±8.37 years old. 25 males and 15 females were grouped into the control group, aged 53-72 years old, with an average of 62.31±8.23 years old. There was no significant difference in the general information of the two groups, which were comparable (Table-I).

**Table I:** Comparative analysis of general information between the experimental group and the control group (X̅ ±S) n=40

Indexes	Experimental group	Control group	t/χ^2^	P
Age	60.15±8.37	62.31±8.23	1.16	0.25
Male (%)	26(65%)	25(62.5%)	0.05	0.82
Hypertension (%)	15(37.5%)	12(30%)	0.50	0.48
Diabetes (%)(%)	13(32.5%)	11(27.5%)	0.24	0.63
Smoking history (%)	23(57.5%)	25(62.5%)	0.21	0.65
History of alcohol abuse (%)	8(20%)	12(30%)	1.07	0.30
Family history (%)	6(15%)	7(17.5%)	0.09	0.76

***Note:*** The underlying diseases of some patients coexist with many diseases, and the one with the largest influence shall prevail, P>0.05.

### Ethical approval:

The study was approved by the Institutional Ethics Committee of Affiliated Hospital of Hebei University (No.: 20200567; date: 18 June, 2020) from March 18, 2020 to October 18, 2020, and written informed consent was obtained from all participants.

### Inclusion criteria:

1. Patients meeting the diagnostic criteria for acute ST-segment elevation myocardial infarction^7^;
2. Patients who have undergone emergency PCI within 12 h of onset;
3. Patients with complete case data;
4. Patients or their family members who agree with the study scheme and sign the consent form and are capable of cooperating with the study.

### Exclusion criteria:

1. Patients with a history of myocardial infarction;
2. Patients with previous arrhythmias such as atrial fibrillation, multiple arrhythmias and other diseases;
3. Patients complicated with malignancies, autoimmune diseases, infections, or inflammatory diseases that affected the study results;
4. Patients with serious heart, liver and renal insufficiency that cannot be satisfactorily controlled;
5. Patients who have recently taken drugs such as hormones and immunosuppressants that may affect the study results;
6. Patients complicated with hematologic diseases, hemorrhagic diseases or hemorrhagic tendencies;
7. Patients with non-STEMI and unstable angina pectoris.

All patients were treated with PCI via radial artery approach under local anesthesia after admission. Patients in both groups were given oxygen inhalation, monitoring, and basic medications for myocardial infarction, such as nutritional myocardial drugs, statins, aspirin, nitrates, clopidogrel, and β-blockers. In contrast, patients in the experimental group received tirofiban 10 ug/kg intravenously over 5min immediately before PCI in addition to basic treatment, and then tirofiban 0.1 ug/(kg/min) was pumped via intravenous pump postoperatively for 48 h. Besides, the platelet count and platelet aggregation rate were monitored.

### Observation indexes

1) *Vascular endothelial function:* The high-resolution color Doppler of German Siemens was used to measure endothelial diastolic function (FMD) of the brachial artery before treatment, one week after treatment and one month after return visit, respectively. Methods: The patient was in a supine position. First, the distance between the anterior and posterior intima of the brachial artery (Db) was measured in the quiet state, and then the distance between the anterior and posterior intima of the brachial artery (D1) was repeatedly measured after the pressure was applied to the distal end of the brachial artery with a sphygmomanometer cuff to 300 mmHg. FMD =[(D1-Db)/Db]×100%^8^. Subsequently, 3-5 mL of early morning and fasting venous blood were drawn at admission, one week and one month after treatment, respectively. Serum nitric oxide (NO) and vascular endothelial constrictor factor-1 (ET-1) levels were detected by ELISA method. 2) *Comparative analysis of changes in cardiac function:* Fastening venous blood was drawn from the patients at admission, one week and one month after treatment, and the difference of N-end brain natriuretic peptide (BNP) between the two groups was compared and analyzed. The left ventricular end-diastolic diameter (LVEDD) and left ventricular etion fraction (LVEF) were measured by the high-resolution color Doppler of Germany Siemens at admission, one week and one month after treatment. Coronary blood flow recovery: Conventional echocardiography (ECG) was performed when the patients were admitted to the hospital and 1 week after treatment, and the incidence of ST reduction greater than 70% in the two groups was compared and analyzed, indirectly reflecting the recovery of coronary blood flow. *Comparative analysis of changes in inflammatory cytokines*: The changes in the levels of IL-6, CRP and other inflammatory cytokines in the experimental group and the control group were observed before treatment and 1 week after treatment; 4) Adverse reactions: The incidence of adverse reactions in the two groups within 1 week after medication was compared.

### Statistical analysis

All the data were statistically analyzed by SPSS 20.0 software, and the measurement data were expressed as (X̄± s). Two independent sample t-test was used for inter-group data analysis, repeated measurement analysis of variance was used for intra-group data analysis, and the paired t-test was used for pairwise comparison. χ^2^ was adopted for rate comparison. P<0.05 indicates a statistically significant difference.

## RESULTS

The changes in vascular endothelial function of the two groups before and after treatment are shown in Table-II. The results indicate that there was no significant difference in the levels of FMD, NO and ET-1 between the two groups before treatment. The above indexes in the control group were not significantly changed 1 week and 1 month after treatment compared with before treatment (*p*>0.05), while those in the experimental group improved significantly 1 week and 1 month after treatment compared with before treatment, with a statistically significant difference (*p*<0.05). In addition, FMD and NO in the experimental group were significantly higher than those in the control Group-I week and 1 month after treatment, with a statistically significant difference (*p*=0.00), while ET-1 was significantly lower than that in the control group, with a statistically significant difference (*p*=0.00).

**Table II:** Comparative analysis of vascular endothelial function between the two groups before and after treatment (X̅ ±S)n=40.

Group		Before treatment*	1 week after treatmentΔ	1 month after treatmentΔ	F	P
FMD(%)	Experimental groupΔ	7.39±2.05	12.37±3.76	15.65±3.38	10.22	0.01
	Control group*	7.43±1.97	7.69±2.28	8.02±2.75	1.10	0.27
	t	0.08	6.73	11.07		
	p	0.93	0.00	0.00		
NO (μmol/L)	Experimental groupΔ	32.41±5.28	41.53±6.27	44.76±5.40	9.73	0.00
	Control group*	33.01±4.75	34.77±3.82	35.07±5.25	2.07	0.08
	t	0.53	5.82	8.14		
	p	0.60	0.00	0.00		
ET- 1 (ng/L)	Experimental groupΔ	80.32±12.41	72.18±9.15	65.71±7.25	6.43	0.00
	Control group*	81.07±11.76	78.45±7.49	77.42±6.51	1.73	0.06
	t	0.27	3.35	7.60		
	p	0.78	0.00	0.00		

* p>0.05, Δp<0.05.

The changes in cardiac function indexes before and after treatment of the two groups are shown in Table-III and Table-IV, suggesting that there was no significant difference in BNP, LVEDD, LVEF and other indexes between the two groups before treatment (*p*>0.05). However, above indexes decreased 1 week after treatment and 1 month after treatment, with a statistically significant difference (*p*=0.00). BNP, LVEDD and LVEF in the experimental group were significantly lower than those in the control group at 1 week and 1 month after treatment, with statistically significant differences (*p*<0.05). The incidence of ST-segment fallback > 70% in the experimental group was 72.5% after treatment, which was significantly better than that of 47.5% in the control group, with a statistically significant difference (*p*=0.03).

**Table III:** Comparative analysis of cardiac function indexes between the two groups (X̅ ±S) n=40.

Group		Before treatment*	1 week after treatmentΔ	1 month after treatmentΔ	F	P
BNP(pg/ml)	Experimental groupΔ	3214.58±625.72	1513.37±321.64	643.57±132.07	20.43	0.00
	Control group*	3287.25±636.70	1758.42±309.78	1125.62±213.76	17.36	0.00
	t	0.51	3.47	12.13		
	p	0.61	0.00	0.00		
LVEDD (mm)	Experimental groupΔ	58.57±4.32	53.41±3.06	50.84±4.74	7.62	0.00
	Control group*	58.03±3.87	56.38±3.43	53.41±4.06	5.21	0.00
	t	0.59	4.10	2.60		
	p	0.56	0.00	0.01		
LVEF(%)	Experimental groupΔ	41.23±4.75	46.83±5.24	51.26±4.92	9.22	0.00
	Control group*	43.08±4.22	44.19±4.85	47.25±5.13	3.75	0.00
	t	1.84	2.34	3.57		
	p	0.06	0.02	0.00		

* p>0.05; Δp<0.05.

**Table IV:** Comparative analysis of the recovery of coronary blood flow between the two groups (X̅ ±S) n=40.

Group	Number of cases where ST segment fallback > 70%	Proportion of ST segment fallback > 70%
Experimental group	29	72.5%
Control group	19	47.5%
χ^2^		4.53
P		0.03

p<0.05.

The changes in inflammatory cytokines before and after treatment in the two groups are shown in Table-V, suggesting that CRP, IL-6 and other inflammatory cytokines were significantly increased in the two groups before treatment, with no significant difference (*p*>0.05), and the above indexes decreased after treatment, with a statistically significant difference (*p*=0.00). After treatment, CRP and IL-6 in the experimental group were significantly lower than those in the control group, with a statistically significant difference (*p*=0.00).

**Table V:** Comparative analysis of the levels of inflammatory cytokine between the two groups before and after treatment (X̅ ±S) n=40.

Observation indexes		Before treatment*	After treatmentΔ	t	P
CRP(mg/L)	Experimental groupΔ	57.43±8..67	15.60±3.57	28.22	0.00
	Control groupΔ	59.13±7.27	19.31±4.10	30.17	0.00
	t	0.95	4.32		
	p	0.35	0.00		
IL-6(ng/L)	Experimental groupΔ	16.64±3.76	8.75±2.73	10.74	0.00
	Control groupΔ	16.78±3.85	10.78±2.05	8.70	0.00
	t	0.16	3.76		
	p	0.83	0.00		

* p>0.05, Δp<0.05.

The incidence of adverse drug reactions in the two groups after treatment was compared and analyzed, suggesting that the incidence of adverse reactions in the experimental group was 25%, and that in the control group was 17.5%. The incidence of adverse reactions in the experimental group was higher than that in the control group, but there was no statistical significance (*p*=0.42). (Table-VI)

**Table VI:** Comparative analysis of adverse drug reactions of the two groups after treatment (X̅ ±S) n=40.

Group	Fever	Gastrointestinal reaction	Skin rash	WBC reduction	Incidence
Experimental group	4	2	3	1	10(25%)
Control group	2	3	1	1	7(17.5%)
χ^2^					0.67
P					0.42

p>0.05.

## DISCUSSION

ST-segment elevation myocardial infarction (STEMI), also known as transmural myocardial infarction, is a severe type of acute myocardial infarction (AMI).^9^ AMI is a disease with poor prognosis, which can be attributed to coronary atherosclerosis that causes stenosis or occlusion of the coronary lumen, resulting in damage to the function of myocardial cells, necrosis, and inducing serious complications such as heart failure and arrhythmia.^10^

PCI is currently the most direct and effective clinical treatment for acute STEMI,^11^ which is characterized by rapid onset and effective protection of cardiomyocytes via rapid recovery of blood supply.^12^ To this end, early diagnosis and immediate reperfusion are the most effective means to diminish the area of myocardial ischemia and infarction, thereby reducing the risk of complications and heart failure after STEMI. PCI has been the preferred reperfusion strategy for patients with STEMI. If PCI cannot be performed within a short time after STEMI diagnosis, fibrinolytic therapy should be considered.^13^ However, there are further opinions that^14^ a variety adverse reactions will be caused after PCI. For example, surgical operations will further aggravate coronary artery intima injury, causing micro-embolism, secondary inflammatory reactions and other reactions^15^, and inflammation cytokines (IL-6, CRP, TNF-α, etc.) gathered around the stent may lead to local inflammation and aggravate a series of cardiovascular events such as heart failure, arrhythmia, and restenosis after stent.^16^

Tirofiban can block the binding of coagulation cytokines and platelet glycoproteins (IIb/IIIa), thereby exerting a role of antiplatelet and anticoagulant.^17^ Furthermore, tirofiban has been touted for its efficacy in degrading formed thrombus, reducing microvascular injury and preventing cardiovascular embolism events.^18^ It is considered by Liu et al.^19^ that tirofiban hydrochloride sodium chloride injection combined with cardiovascular intervention has important clinical effects in the treatment of acute myocardial infarction. It is of high application value and is beneficial to effectively improve blood perfusion, reduce the occurrence of adverse cardiac events, and have a favorable effect on the prognosis of patients. It has also been confirmed in the study of Chen et al.^20^ that intracoronary injection of tirofiban can effectively improve myocardial reperfusion in elderly patients with PCI after emergency PCI, which is also very beneficial to the improvement of prognosis. According to the study of Wang et al.^21^, moderate doses of tirofiban have less side effects. Li et al. also believed^22^ that moderate doses of tirofiban would not increase the risk of bleeding in patients. It was confirmed in this study that FMD, NO, ET-1 and other indicators in the experimental group were significantly improved compared with the control Group-I week and 1 month after treatment, with a statistical significance (*p*<0.05). Indexes of LVEDD and LVEF in the experimental group at 1 week and 1 month after treatment were significantly lower than those in the control group, with a statistical significance (*p*=0.00). Moreover, the incidence of ST-segment fallback > 70% in the experimental group was 72.5% after treatment, which was significantly better than that of 47.5% in the control group, with a statistically significant difference (*p*=0.03). There was no statistical significance in the incidence of adverse drug reactions between the two groups after treatment (*p*=0.42).

Excessive levels of inflammatory cytokines^23^ and N-terminal brain natriuretic peptide (BNP)^24^ will have a serious impact on the prognosis of patients with myocardial infarction. Tirofiban has been shown in some studies^25^ to have anti-inflammatory activity, which can reduce further damage caused by flammatory cytokines. It was suggested in our study that CRP and IL-6 in the experimental group were significantly lower than those in the control group after treatment, with a statistically significant difference (*p*=0.00); BNP in the experimental group was significantly lower than those in the control Group-I week and 1 month after treatment, with a statistically significant difference (*p*=0.00). It is proved that tirofiban injection after emergency PCI has a good therapeutic effect on patients with acute myocardial infarction.

### Limitations of the study:

Nevertheless, deficiencies can still be seen in this study: fewer samples are included in the study, long-term follow-up results are lacking, and other antiplatelet aggregation agents are not compared with tirofiban due to small sample size and other reasons. In view of this, proactive countermeasures are being taken to accumulate cases, further extend the follow-up time, and gradually increase the comparative analysis of other antiplatelet drugs in some cases, so as to conduct a more objective evaluation of the long-term effects of this treatment regimen.

## CONCLUSIONS

To put it in a nutshell, tirofiban injection after emergency PCI is a beneficial treatment regimen for patients with STEMI. With such a treatment regimen, cardiac function and vascular endothelial function of patients can be significantly improved, coronary blood supply will be ameliorated, inflammatory cytokines can be reduced, and no significant increase can be seen in the incidence of adverse reactions.

### Authors’ Contributions:

**SXK & XMM:** Designed this study and prepared this manuscript,and are responsible and accountable for the accuracy or integrity of the work.

**XMM:** Collected and analyzed clinical data.

**JL:** Significantly revised this manuscript.
